# Whole-Genome Metalloproteases in the Wheat Sharp Eyespot Pathogen *Rhizoctonia cerealis* and a Role in Fungal Virulence

**DOI:** 10.3390/ijms231810691

**Published:** 2022-09-14

**Authors:** Feilong Guo, Lijun Pan, Hongwei Liu, Liangjie Lv, Xiyong Chen, Yuping Liu, Hui Li, Wenwu Ye, Zengyan Zhang

**Affiliations:** 1 Key Laboratory of Biology and Genetic Improvement of Triticeae Crops, Ministry of Agriculture and Rural Affairs of the People’s Republic China, The National Key Facility for Crop Gene Resources and Genetic Improvement, Institute of Crop Sciences, Chinese Academy of Agricultural Sciences, Beijing 100081, China; 2 Institute of Cereal and Oil Crops, Hebei Academy of Agriculture and Forestry Sciences, Shijiazhuang 050035, China; 3 The Key Laboratory of Plant Immunity, Department of Plant Pathology, Nanjing Agricultural University, Nanjing 210095, China

**Keywords:** cell death, metalloproteases, *Rhizoctonia cerealis*, virulence, wheat (*Triticum aestivum* L.)

## Abstract

*Rhizoctonia cerealis* is the causal agent of sharp eyespot, a devastating disease of cereal crops including wheat. Several metalloproteases have been implicated in pathogenic virulence, but little is known about whole-genome metalloproteases in *R. cerealis*. In this study, a total of 116 metalloproteases-encoding genes were identified and characterized from the *R. cerealis* Rc207 genome. The gene expression profiles and phylogenetic relationship of 11 MEP36/fungalysin metalloproteases were examined during the fungal infection to wheat, and function of an upregulated secretory MEP36 named RcFL1 was validated. Of 11 MEP36 family metalloproteases, ten, except RcFL5, were predicted to be secreted proteins and nine encoding genes, but not *RcFL5* and *RcFL2*, were expressed during the *R. cerealis* infection process. Phylogenetic analysis suggested that MEP36 metalloproteases in *R. cerealis* were closely related to those of *Rhizoctonia solani* but were remote to those of *Bipolaris sorokiniana*, *Fusarium graminearum*, *F. pseudograminearum*, and *Pyricularia oryzae*. Expression of RcFL1 was significantly upregulated during the infection process and induced plant cell death in wheat to promote the virulence of the pathogen. The MEP36 domain was necessary for the activities of RcFL1. Furthermore, RcFL1 could repress the expression of wheat genes coding for the chitin elicitor receptor kinase TaCERK1 and chitinases. These results suggest that this MEP36 metalloprotease RcFL1 may function as a virulence factor of *R. cerealis* through inhibiting host chitin-triggered immunity and chitinases. This study provides insights on pathogenic mechanisms of *R. cerealis*. *RcFL1* likely is an important gene resource for improving resistance of wheat to *R. cerealis* through host-induced gene silencing strategy.

## 1. Introduction

Bread wheat (*Triticum aestivum* L.) is one of the most important staple crops worldwide [1]. However, yield and grain quality of wheat often are negatively impacted by fungal diseases [2]. The necrotrophic fungus *Rhizoctonia cerealis* van der Hoeven [3] is the major causal agent of sharp eyespot, a destructive stem-base disease of wheat [4,5]. Sharp eyespot can reduce yields of wheat in many regions of the world, including Asia, Oceania, Europe, North America, and Africa [6,7,8,9]. In addition, the fungus *R. cerealis* can also infect other important economical crops and bio-energy plans, including barley (*Hordeum vulgare*), rye (*Secale cereale*), sugar beet (*Beta vulgaris*), cotton (*Gossypium* spp.), potato (*Solanum tuberosum*), and turfgrasses [10,11]. To control wheat sharp eyespot, it is vital to identify host resistance genes, mine important virulence factors of the pathogen *R. cerealis* and study mechanisms underlying the fungus–host interactions. Thus, we have recently completed the genome sequencing and assembling/annotation of the wheat sharp eyespot pathogen *R. cerealis* strain Rc207 (Zengyan Zhang’s lab, GSA no. CRA007128) to establish databases for further study on pathogen–host plant interactions and the fungal virulence factors. 

Benhamou et al. demonstrated *Rhizoctonia* cell walls to be high in chitins so that hydrolysis by a bean chitinase is antifungal [12]. In plants, chitinases can degrade chitin of the fungal cell wall, rendering the cell wall unstable. Chitinases also have the secondary effect to release chitin, which will trigger a defense response because they are acting as a pathogen- or microbe-associated molecular pattern (PAMP/MAMP). Perception of PAMPs/MAMPs by pattern-recognition receptors initiates defense responses leading to basal resistance in plants, including the reinforcement of plant cell walls and the production of defense molecules [13]. Due to the crucial role of chitin for the activation of the plant immune system, fungi have evolved complicated mechanisms, secrete LysM domain-containing proteins, metalloproteases, and secretory polysaccharide deacetylase, to suppress plant chitinases and stop the chitin-mediated activation of the plant’s immune system [14,15,16,17,18]. For instance, the *Fusarium verticillioides* fungalysin metalloprotease Fv-cmp can cleave the maize class IV chitinases ChitA and ChitB during maize ear rot [17]. In the soil-borne fungus *Verticillium dahlia,* a secretory polysaccharide deacetylase VdPDA1 is required for full virulence of the pathogen through preventing host chitin-triggered immunity [18].

Metalloproteases (MEPs) are multiple families of hydrolytic enzymes; the majority is zinc-dependent due to two zinc-binding histidine residues in a HEXXH motif [17,19,20]. A few studies on functions of several MEP36 (fungalysin) and the MEP35 (deuterolysin) as well as MEP43 metalloproteinases in pathogens have been reported. For example, the fungus *Fusarium oxysporum* f. sp. lycopersici produces a fungalysin (MEP36) named FoMEP1, which acts in concert with the serine protease FoSep1 to cleave chitinases produced by tomato. The double deletion mutant of FoMep1 and FoSep1 can reduce virulence of *F. oxysporum* on tomato [21]. The M36 metalloprotease Cgfl in *Colletotrichum graminicola* has been demonstrated to have the role in the degradation of chitinases like other fungi fungalysins, and to enhance virulence of this pathogenic fungus causing the maize anthracnose disease [22]. Avr-Pita, a MEP35 metalloprotease of the rice blast fungus *Pyricularia oryzae* (*Magnaporthe oryzae*), functions as an effector and is recognized by the rice cytoplasmic resistance protein Pi-ta [23]. In *R. cerealis,* MEP43 metalloprotease RcMEP1 was shown to be required for the pathogen virulence [24]. Moreover, nine metalloproteases in *Phytophthora infestans* were reported to be potential pathogenicity factors in *P. infestans* [25]. However, little is known about whole-genome metalloproteases in *R. cerealis*.

In this study, we identified a total of 116 metalloprotease-encoding genes from the *R. cerealis* Rc207 genome, and examined their transcriptional profiles in time-course during the fungal infection to wheat. Since almost among 11 genes coding MEP36 metalloproteases (fungalysins) in the *R. cerealis* genome, nine genes, but not *RcFL5* and *RcFL2*, were expressed and ten proteins, except RcFL5, were predicted as secreted proteins, we analyzed the sequences of MEP36 metalloproteases and their genes’ structures, phylogenetic relationship, and transcriptional profiles during *R. cerealis* infection to wheat. Notably, we investigated the functional roles of the MEP36 metalloprotease RcFL1 and its MEP36 domain in the fungal virulence to wheat, and the results suggested that RcFL1 acted as a virulence factor through inducing plant-cell death and inhibiting the expression of *chitinases* (*TaChit3* and *TaChitIV*) and chitin elicitor receptor kinase-encoding gene (*TaCERK1*) in the treated wheat. 

## 2. Results

### 2.1. Genome-Wide Characterization of Metalloproteases Based on R. cerealis Genome Assembly

The recently-constructed proteases database MEROPS currently contains over 4000 entries, and MEROPS classification is an excellent tool for categorizing proteases deduced from genome annotations and predicting functions of validated proteases [20,25]. Herein, the MEROPS database (release 12.4) [20] was deployed to identify genome-wide metalloproteases from the high-quality genome assembly of *R. cerealis* Rc207. The results showed that the Rc207 genome contained a total of 116 genes encoding metalloproteases, belonging to 24 families (Table S1). In these 24 metalloprotease families, MEP24, MEP28, and MEP36 families are particularly richer in *R. cerealis* Rc207. Based on the presence of a typical signal peptide, 34 metalloproteases in *R. cerealis* Rc207 genome belong to secretory proteins. Among them, 3, 3, 9, 8, 10, and 1 secretory proteins belong to the MEP1, MEP14, MEP28, MEP35, MEP36, and MEP64 families, respectively (Table S1). Furthermore, in *R. cerealis* Rc207, 32 metalloproteases are also annotated as pathogen–host interaction (PHI) proteins, which may play significant roles in pathogen infection (Table S1). Although certain researchers annotated candidate effectors with a signal peptide-containing protein pipeline [26], experimentally-validated effectors/virulance factors tend to be cysteine-rich (≥4) [27]. Our analysis indicated that these 116 metalloproteases included 93 cysteine-rich (the number of cysteine ≥ 4) metalloproteases (Table S1). These secreted, cysteine-rich metalloproteases likely function as virulence factors.

To further investigate the sequence similarity between metalloproteases in the *R. cerealis* Rc207 genome, OrthoFinder v2.5.1 software (https://github.com/davidemms/OrthoFinder/releases, accessed on 30 November 2020) was used to group all metalloprotease sequences into ortholog groups (OGs). These 116 metalloproteases from *R. cerealis* Rc207 were clustered in 65 OGs by OrthoFinder (Table S1). Then, we analyzed the OG distribution of the metalloproteases in the *R. cerealis* Rc207 genome, and results showed that 41 OGs are specific, containing only one metalloprotease, and the largest OG present in the data set is OG6_103356 with nine metalloproteases from the MEP28 family, which are able to release a variety of N-terminal amino acids (Table S1).

### 2.2. Characterics of MEP36 Metalloproteases and Their Encoding Genes’ Structures

**Figure 1:**
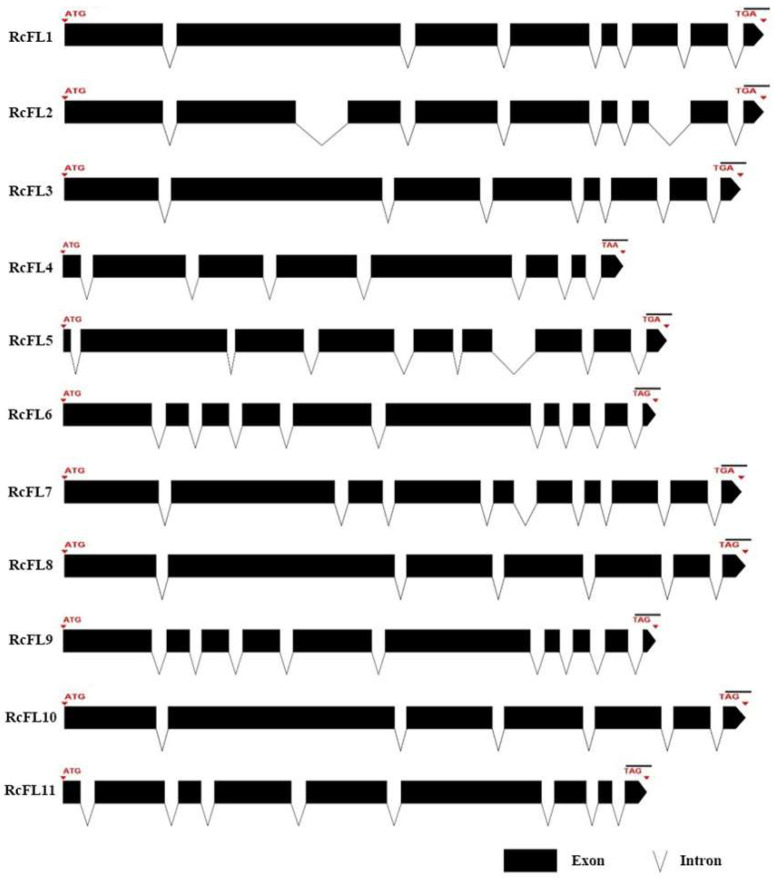
Structures of *MEP36*-contained metalloprotease genes of *R. cerealis*. Exons and introns are indicated by black boxes and lines.

We focused on MEP36 domain-containing metalloproteases, possessing HEXXH motifs characteristic of Zn-binding metalloproteases, since almost all MEP36 metalloproteases in *R. cerealis* Rc207 genome are predicted as secreted proteins, each with a signal peptide in the N-terminal. In previous studies, MEP36 metalloproteases have been named as fungalysins [17,22]. Thus, these 11 *MEP36* genes in *R. cerealis* are designated as *RcFL1–RcFL11* (Table 1). By means of analysis using DNAMAN software, we found that the metalloprotease gene *RcFL4* possesses the shortest coding sequence size with 1788 bp, and the coding sequence sizes of *RcFL8* and *RcFL10*, with 2346 bp, are the longest. Multiple sequence alignment results showed that the identity among the coding sequences of the 11 *RcFLs* genes was 56.95% (Figure S1). All the 11 *MEP36* metalloprotease genes in *R. cerealis* had different intron–exon patterns in relation to both the position and number of introns. Based on the number of introns, these metalloproteases were segregated into four patterns, pattern1 (*RcFL8* and *RcFL10*), pattern 2 (*RcFL1*, *RcFL3*, and *RcFL4*), pattern 3 (*RcFL2*, *RcFL5*, and *RcFL11*), and pattern 4 (*RcFL6*, *RcFL7*, and *RcFL9*), containing 6, 7, 8, and 9 introns, respectively. Furthermore, significant differences in size (28–875 bp) between the exons were observed. These results suggested that these *MEP36* metalloprotease genes had variable and complex gene structures (Figure 1).

**Figure 2:**
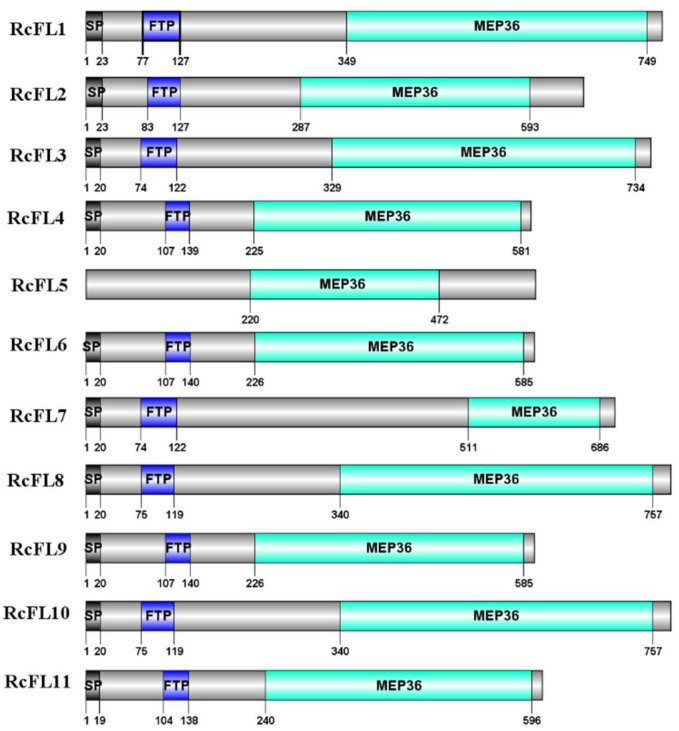
Predicted functional domains present in the MEP36 family metalloproteases of *R. cerealis*. SP: Signal peptide, FTP: Fungalysin/Thermolysin Propeptide Motif. Green: MEP36 domain.

The Pfam software was used to predict the domains of these RcFLs proteins, and the results showed that all the 11 RcFL proteins, RcFL1-RcFL11, contained the MEP36 domain. With the exception of RcFL5, 10 remaining RcFLs all contained a signal peptide with a length of 19–23 amino acid (aa) residues and a Fungalysin/Thermolysin Propeptide Motif (FTP) with a length of 32–50 aa residues (Figure 2). Thus, expect for RcFL5, the 10 remaining *R. cerealis* metalloproteases, namely RcFL1–RcFL4 and RcFL6–RcFL11, were predicted to be secreted proteins based on the presence of a typical signal peptide (Table 1). Using DNAMAN software, we found that the shortest metalloprotease protein, RcFL4, consisted of 595 aa residues, and the longest metalloprotease proteins, RcFL8 and RcFL10, had 781 aa residues. The lengths of the MEP36 domains across RcFL1–RcFL11 ranged from 175 aa (RcFL7) to 417 aa (RcFL8 and RcFL10) residues (Figure 2). The predicted molecular weights of all the metalloprotease proteins ranged from 63.38 kD to 86.13 kD and their isoelectric points (pIs) ranged from 5.20 to 6.68, respectively (Table 1). Multiple sequence alignment results showed that the identity among the amino acid sequences encoded by the 11 *RcFLs* genes was 49.70% (Figure S2).

### 2.3. Phylogenetic Analysis of MEP36 Metalloproteases among R. cerealis and Other Phytopoathogens

**Figure 3:**
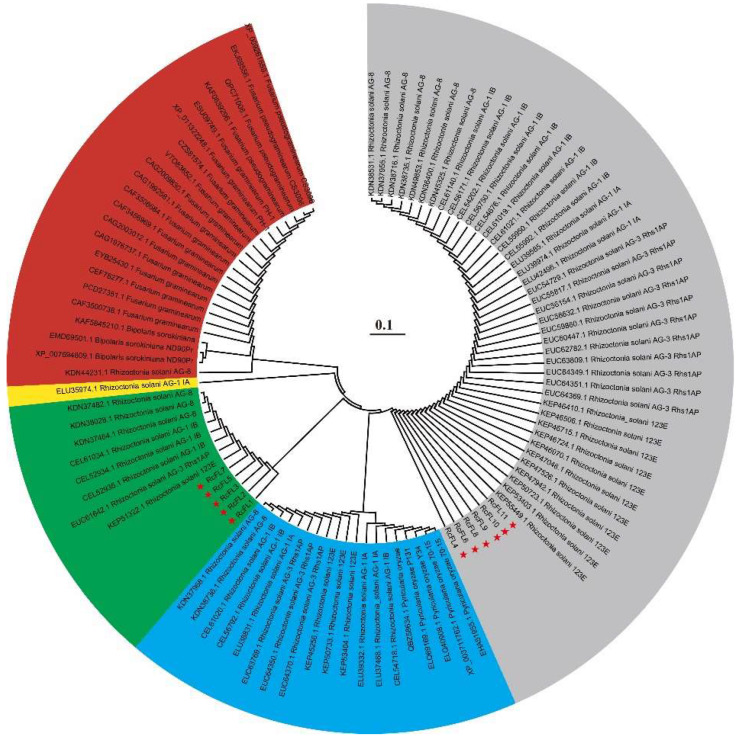
Phylogenetic relationships among MEP36 metalloproteases from *R. cerealis* and other fungal plant-pathogens. The phylogenic tree was constructed by Mega 7.0 using the neighbor-joining method (parameters: 1000 bootstraps). Grey represents group I; Blue represents group II; Green represents group III; Yellow represents group IV; Red represents group V. These stars represent RcFLs proteins from *R. cerealis*.

To examine the relationship between the MEP36 metalloproteases of *R. cerealis* (RcFLs) and those from other plant–pathogens, we constructed a phylogenetic tree based on the multiple amino acid sequence alignment on the 11 RcFLs metalloproteases and 91 MEP36 metalloproteases from other fungal plant–pathogens, namely *Bipolaris sorokiniana* (ND90Pr), *Fusarium graminearum* (PH-1), *Fusarium pseudograminearum* (CS3096), *Pyricularia oryzae* (P131, Y34, 70-15), and *Rhizoctonia solani* (AG1-IA, AG1-IB, 123E, AG-3 Rhs1AP, and AG-8) (Figure 3).

The resulting phylogenetic tree divided these MEP36 metalloproteases into five groups (Figure 3). The Group I (grey) was constituted by RcFL4, RcFL6, RcFL8, RcFL9, RcFL10, and RcFL11 from *R. cerealis* Rc207 with seven MEP36 metalloproteases from *R. solani* AG-8 (KDN45325.1, KDN36400.1, KDN49853.1, KDN38735.1, KDN38716.1, KDN38531.1, and KDN37955.1), nine MEP36 metalloproteases from *R. solani* AG-1 IB (CEL55992.1, CEL55950.1, CEL61021.1, CEL61019.1, CEL54676.1, CEL56750.1, CEL54202.1, CEL56171.1, and CEL61140.1), three MEP36 metalloproteases from *R. solani* AG-1 IA (ELU39585.1, ELU39974.1, and ELU42496.1), eleven MEP36 metalloproteases from *R. solani* AG-3 Rhs1AP (EUC64369.1, EUC64351.1, EUC64349.1, EUC63809.1, EUC62782.1, EUC60447.1, EUC59860.1, EUC58632.1, EUC56154.1, EUC55817.1, and EUC54729.1), and eleven MEP36 metalloprotease from *R. solani* 123E (KEP55449.1, KEP53403.1, KEP50723.1, KEP47942.1, KEP47526.1, KEP47048.1, KEP46970.1, KEP46724.1, KEP46715.1, KEP46506.1, and KEP46410.1). Group III (Green) contained RcFL1, RcFL2, RcFL3, RcFL5, RcFL7, three MEP36 metalloproteases from *R. solani* AG-8 (KDN37482.1, KDN38028.1, and KDN37484.1), three MEP36 metalloproteases from *R. solani* AG-1 IB (CEL61034.1, CEL52934.1, and CEL52935.1), an MEP36 metalloprotease from *R. solani* AG-3 Rhs1AP (EUC61642.1), and a MEP36 metalloprotease from *R. solani* 123E (KEP51322.1). None of the *R. cerealis* RcFLs proteins were clustered in Group II (blue), IV (yellow), and V (red). 

### 2.4. Gene Expression Analysis of Metalloproteases during R. cerealis Infection Process to Wheat

To investigate how the metalloprotease genes of fungal transcriptional reprogramming occurs during *R. cerealis* infection to wheat, we performed RNA-sequencing to investigate transcript dynamics of the metalloprotease genes in the *R. cerealis* Rc207 strain at five infection time-points (18, 36, 72, 96, and 240 h after inoculation, hai) to wheat and in culture (vitro) mycelia. The transcriptomes showed that 82, 82, 98, 116, and 116 genes were expressed at 18, 36, 72, 96, and 240 hai, suggesting there was a temporal sequence leading to full expression of all genes (Table S2, Figure S3). Compared with in vitro, a total of 6, 4, 13, 19, and 19 genes were significantly upregulated (log_2_fold-change > 1, FDR *P* < 0.05) at 18, 36, 72, 96, and 240 hai, respectively (Table S2, Figure S3). 

**Figure 4:**
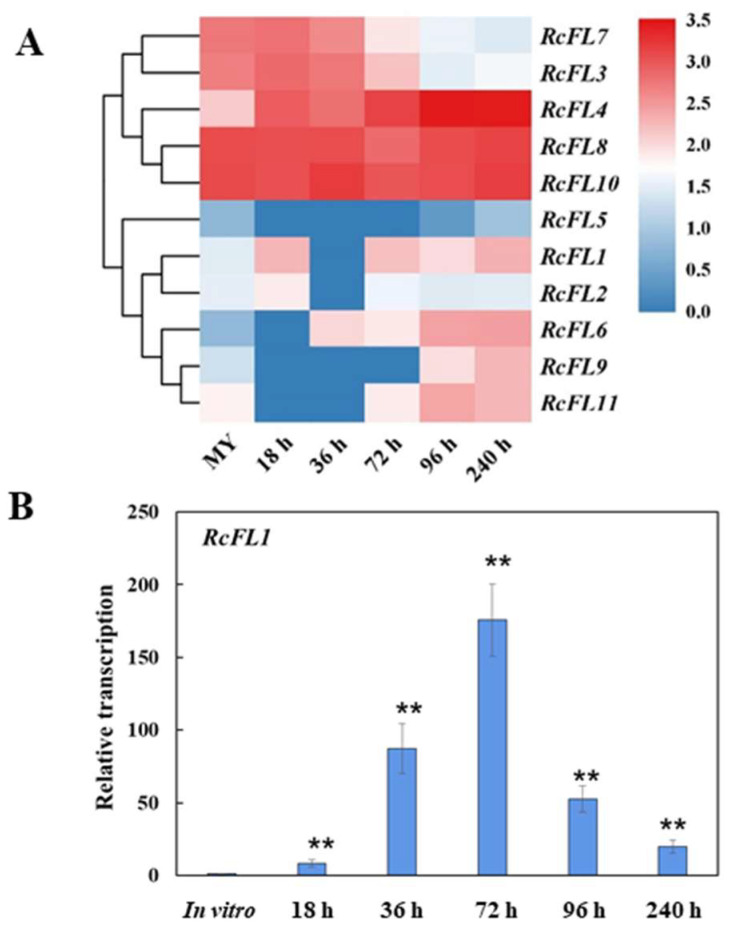
Expression patterns of MEP36 metalloproteases *(RcFLs*) genes of *R. cerealis*. (**A**) Heat map showing expression levels of *RcFLs* of *R. cerealis* Rc207 during wheat infection. The color bar represents the log2 of (FPKM+1) value; (**B**) RT-qPCR assay showing the expression levels of the *RcFL1* gene in *R. cerealis* Rc207 during different infection stages. The *R. cerealis Actin* gene was used as an internal control to normalize the data. Standard error (SE) bar was calculated based on three technical replicates and significance was assessed using Student’s *t*-tests (**, *p* < 0.01).

Furthermore, we investigated the gene expression profiles of the 11 *MEP36* metalloproteases in *R. cerealis*. Their RNA-sequencing results were shown in the heat map, and nine encoding genes were expressed during *R. cerealis* infection process to wheat (Figure 4A). Among them, the transcript levels of the *RcFL1* (*Rc_11192.1*) were significantly up-regulated during *R. cerealis* infection process (18, 72, 96, and 240 hai) compared to in vitro fungal mycelia (Figure 4A). The RT-qPCR analysis indicated that the transcript abundances of the *RcFL1* (*Rc_11192.1*) were markedly upregulated during the *R. cerealis* infection process (18, 36, 72, 96, and 240 hai) compared to in vitro fungal mycelia (Figure 4B), peaked at 72 hai. Hereafter, we further investigated the functional role of the *RcFL1* in *R. cerealis* infection to wheat. 

### 2.5. RcFL1 Induces Necrosis and Plant-Cell Death in Leaves of Wheat and Nicotiana benthamiana 

**Figure 5:**
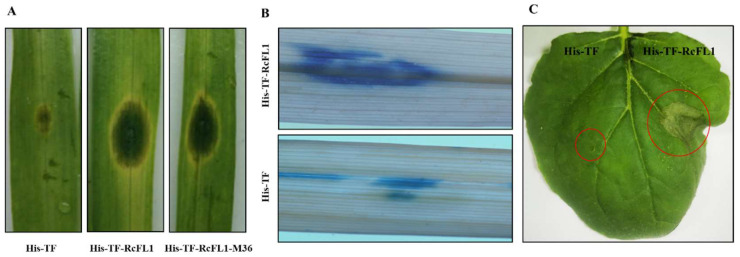
RcFL1 induces plant cell death shown as leaf lesions in wheat and *N. benthamiana*. (**A**) RcFL1 and its M36 domain cause lesions in infiltrated leaves of the susceptible-*R. cerealis* wheat cv. Wenmai 6; (**B**) trypan blue staining of wheat cv. Wenmai 6 leaves infiltrated with His-TF-RcFL1 or His-TF. Dead wheat leaf cells were stained by trypan blue. (**C**) RcFL1 can induce the cell death in *N. benthamiana*. Each heterologously-expressed protein, His-TF-RcFL1, His-TF-RcFL1-MEP36 or His-TF, was infiltrated in 25 µL (concentration 5 µM/L). Six leaves were used in each experiment and repeated three times. Red circles represent the sites of His-TF and His-TF-RcFL1 protein infiltration or the cell-death occurring.

To investigate role of *RcFL1* in virulence of *R. cerealis*, we examined whether RcFL1 full-length protein and the truncated version containing MEP36 domain (no. 349–749 aa, RcFL1-MEP36) can induce the cell death of wheat or *N. benthamiana* leaves. To obtain purified RcFL1 and RcFL1-MEP36 proteins, the full *RcFL1* or the *RcFL1-MEP36* peptide-encoding sequences were separately sub-cloned in fusion to the His-TF tag of the pCOLD Trigger Factor (TF) vector, generating two recombinant protein vectors pHis-TF-RcFL1 and pHis-TF-RcFL1-MEP36. After induction by 0.5 mM isopropyl-β-d-thiogalactoside at 16 °C, the resulting pHisTF-RcFL1 and pHis-TF-RcFL1-MEP36 as well as the control His-TF proteins were highly expressed in *E. coli* DE3, respectively. After purification using Ni+ resin and elution with imidazole buffer, these proteins were examined through SDS-PAGE (Figure S4). Subsequently, the purified His-TF-RcFL1, His-TF-RcFL1-MEP36, or His-TF proteins, each 25 µL (concentration 5 µM/L), were individually infiltrated into detached leaves of the *R. cerealis*-susceptible wheat cv. Wenmai 6. As a result, compared to His-TF (CK), the His-TF-RcFL1 (expressing the full RcFL1 protein) and His-TF-RcFL1-MEP36 (expressing the M36 domain of RcFL1) were able to trigger more obvious necrosis and plant cell death on the infiltrated leaves of susceptible wheat cv. Wenmai 6 (Figure 5A). Additionally, the results of trypan blue staining, which detects plant cell death, showed that, compared with those treated by His-TF control protein, the stain sizes in wheat leaves with His-TF-RcFL1 were larger and more marked (Figure 5B). These results suggest that RcFL1 is able to induce plant cell death and that the MEP36 domain is required for the cell death-inducing activity of this metalloprotease. Cell death induction by His-TF-RCFL1 was reproduced also in tobacco leaves (Figure 5C).

### 2.6. RcFL1 Protein Is Required for R. cerealis Infection to Wheat

**Figure 6:**
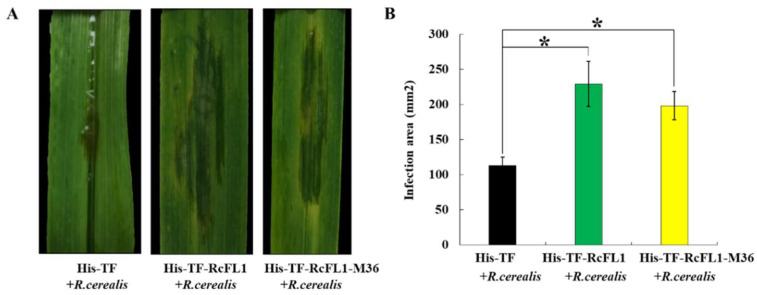
RcFL1 protein and its MEP36 domain-containing peptide contribute to virulence of *R. cerealis*. (**A**) His-TF-RcFL1 and His-TF-RcFL1-MEP36 enhanced infection area of *R. cerealis* in wheat leaves. Heterologously-expressed protein, His-TF-RcFL1, His-TF-RcFL1-MEP36, or His-TF; each was infiltrated in 25 µL (concentration 5 µM/L). Pictures of the lesions were taken at three days post inoculation with the fungus and the lesion area was measured; (**B**) the lesion area/infection was measured after *R. cerealis* Rc207 liquid mycelia inoculation for three days on leaves infiltrated with heterologously-expressed RcFL1, RcFL1-MEP36 in 25 µL (concentration 5 µM/L). Six leaves were used in each experiment and repeated three times. Asterisk * indicates significant difference between His-TF-RcFL1 or His-TF-RcFL1-MEP36 treatment and His-TF treatment (*t*-tests, *p* < 0.05).

To further examine the virulence roles of RcFL1 protein or its MEP36 domain-containing peptide (RcFL1-MEP36), the control His-TF, RcFL1, or RcFL1-MEP36 proteins were individually infiltrated into leaves of a *R. cerealis*-susceptible wheat cv. Wenmai 6 for six hours, and then the level surfaces were further inoculated with liquid hypha of *R. cerealis* Rc207. After three days, the water-soaking (disease lesion) areas in these wheat leaves were assessed. As a result, the larger disease lesion sizes were present in His-TF-RcFL1 and His-TF-RcFL1-MEP36 infiltrated leaves compared to those infiltrated with the His-TF control (Figure 6A). The statistical analysis also indicated the significantly different lesions occurred between the His-TF-RcFL1/ His-TF-RcFL1-MEP36 and the His-TF (Figure 6B). These results suggested that both RcFL1 and its MEP36 domain peptides contribute to virulence of *R. cerealis* and the fungal infection to wheat. 

### 2.7. RcFL1 Represses the Expression of TaCERK1 (a Chitin Elicitor Receptor Kinase) and Chitinases in Wheat

**Figure 7:**
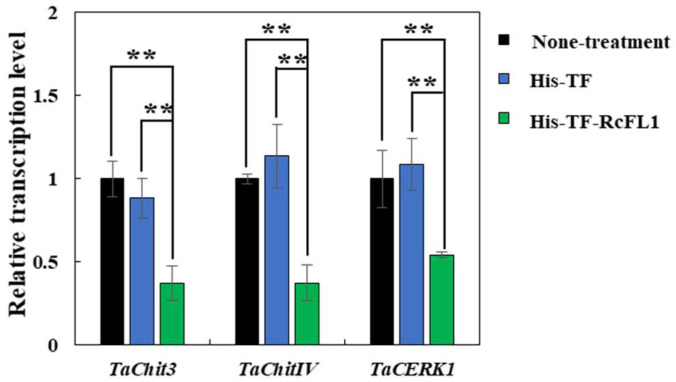
The RcFL1 protein treatment decreases the transcripts of wheat chitinases and receptor-like kinase in infiltrated leaves. RNAs were extracted from wheat leaves at 3 d post treatment with His-TF-RcFL1 or the His-TF (CK) in 25 µL at a concentration of 5 µM/L and none-treatment. The wheat *Actin* gene was used as an internal control to normalize the data. SE was calculated based on three technical replicates. ** indicates significant differences (*p* < 0.01, *t*-test) between His-TF-RcFL1 and the His-TF infiltrated leaves.

To explore if RcFL1 functional role is related to the chitin elicitor pathway or chitinases in wheat, RT-qPCR was deployed to examine transcript levels of *TaCERK1 (TraesCS6D01G403200.1)*, a wheat chitin elicitor receptor kinase CERK1, and two chitinase-coding genes (*TaChit3* and *TaChitIV*) in the susceptible wheat cv. Wenmai 6 leaves infiltrated with His-TF-RcFL1 or the His-TF control. The results showed that the expression levels of *TaCERK1, TaChit3* and *TaChitIV* were significantly decreased by the RcFL1 protein in the His-TF-RcFL1 infiltrated wheat leaves compared to His-TF infiltrated wheat leaves and healthy wheat leaves (Figure 7), suggesting that RcFL1 repressed the expression of the kinase gene *TaCERK1* and *chitinases* (*TaChit3* and *TaChitIV*) in wheat.

## 3. Discussion

Metalloproteinases, in which zinc is an essential metal ion for the catalytic activity, have been identified with the pathogenicity and virulence of pathogenic fungi [17,19,20,21,22,23,24,25,28]. In this study, a total of 116 metalloproteases were identified from the advanced whole-genome sequencing data of the wheat sharp eyespot pathogen *R*. *cerealis* Rc207. They were divided into 24 protein families, including 34 putative secretory metalloproteases with a predicted signal peptide. In the plant pathogen *P. infestans*, 91 metalloproteases were identified and divided over 21 protein families, including 25 metalloproteases with a predicted signal peptide or signal anchor [25]. Researchers reported that secreted proteins with a signal peptide often are candidate effectors or are important for virulence of pathogens [24,25,26,27,29,30]. In the *R. cerealis* genome, the MEP1, MEP14, MEP28, MEP35, MEP36, and MEP64 families contain 3, 3, 9, 8, 10, and 1 secretory metalloproteases, respectively. Previously, only 5 MEP35 proteins were identified from next-generation genome sequences of *R*. *cerealis* Rc207. These results suggest that the advanced whole-genome assembly of the *R*. *cerealis* Rc207 is high-quality.

The current analysis showed that ten (90.91%) of 11 MEP36 metalloproteases are predicted to be secreted proteins. MEP36 family metalloproteases all harbor a HEXXH motif, in which two histidine residues function as the first and the second zinc ligand. Several MEP36 metalloproteases have been shown to play important roles in pathogenicity/virulence of other pathogens [17,21,22]. Hereafter, our further investigations focused on MEP36 metalloproteases in *R. cerealis*. With the exception of RcFL5, 10 remaining RcFLs all contained a signal peptide with a length of 19–23 aa residues. Nine encoding genes, but not *RcFL5* and *RcFL2*, were expressed during *R. cerealis* infection process to wheat. Such facts may imply that RcFL1-RcFL11, but not RcFL5 and RcFL2, may play roles in the fungal infection. Phylogenetic analysis indicated that RcFL4, RcFL6, RcFL8, RcFL9, RcFL10, and RcFL11 from *R. cerealis* Rc207 with 41 MEP36 metalloproteases from *R. solani* AG-8, *R. solani* AG-1 IB, *R. solani* AG-1 IA, *R. solani AG-3* Rhs1AP, and *R. solani* 123E were clustered into the group I, while RcFL1, RcFL2, RcFL3, RcFL5, RcFL7, with three MEP36 metalloproteases from *R. solani* AG-8, three MEP36 metalloproteases from *R. solani* AG-1 IB, a MEP36 metalloproteases from *R. solani* AG-3 Rhs1AP, and a MEP36 metalloprotease from *R. solani* 123E were clustered into the Group III. None of the *R. cerealis* MEP36 proteins were clustered with other MEP36 metalloproteases of plant pathogens *B. sorokiniana* (ND90Pr), *F. graminearum* (PH-1), *F. pseudograminearum* (CS3096), and *P. oryzae*. These data suggest that MEP36 metalloproteases in *R. cerealis* were more closely-related to those of *R. solani* but were remote to those of *B. sorokiniana* (ND90Pr), *F. graminearum* (PH-1), *F. pseudograminearum* (CS3096), and *P. oryzae* (P131, Y34, 70-15).

Necrotrophic fungal pathogens utilize an array of effectors to induce plant cell death which may facilitate the growth of the necrotrophic pathogens [27,30]. Our previous study showed that the secretory M43 metalloprotease of *R. cerealis* are able to induce plant cell-death and may act as a virulence factor of *R. cerealis* [24]. Here, gene profile analyses showed that the transcript abundance of the fungal MEP36 gene *RcFL1* was markedly upregulated during the *R. cerealis* infection process (18, 36, 72, 96, and 240 hai) compared to the fungal grown *in* *culture*. Furthermore, the functional analyses demonstrated that the secretory *R. cerealis* MEP36 metalloprotease RcFL1 could induce plant cell-death and necrotic lesions, and promoted the fungal virulence. Moreover, our analyses suggest that the MEP36 domain is necessary for the function of the metalloprotease RcFL1 in the virulence of *R. cerealis*.

Chitin is a key component of the cell walls of pathogenic fungi, such as *Rhizoctonia* isolates [12]. Plants have evolved chitin-mediated defense responses againstpathogenic fungi, including generation of chitinases and chitin-triggered immunity [12,18,22,23,31,32]. Meanwhile, plant pathogenic fungi can generate various enzymes and proteins, such as polysaccharide deacetylase and metalloproteases, to inhibit host plant chitin-triggered immunity or directly degrade chitinases [17,18,22]. For instance, VdPDA1, a secretory polysaccharide deacetylase in the soil-borne fungus *Verticillium dahlia,* is required for full virulence of the pathogen through preventing host chitin-triggered immunity [18]. The fungalysin/MEP36 metalloprotease from maize pathogen *F. verticilloides* could completely cleave the classs IV chitinase ChitA from maize [17]. Interestingly, the current research indicated that the *R. cerealis* MEP36 metalloprotease RcFL1 could repress the expression of *chitinases* (*TaChit3* and *TaChitIV*) and gene coding for the chitin elicitor receptor kinase TaCERK1 in host wheat. The data imply that the MEP36 metalloprotease RcFL1 functions as a virulence factor of *R. cerealis* possibly through preventing the host chitin-triggering immunity and reduced transcript accumulation of chitinases. It is very interesting to determine experimentally the detailed role of RcFL1 in the loss of this MAMP system in future. Taken together, the fungal-pathogen MEP36 metalloprotease RcFL1 can promote plant cell death and turn down host defense measure to help *R. cerealis* infect wheat.

## 4. Materials and Methods

### 4.1. Plant Materials, Fungal Strain, Growth Conditions, and Primers

Sharp eyespot-susceptible wheat cultivar Wenmai 6 plants were grown in a greenhouse under a 13-h light (~22 °C)/11-h dark (~10 °C) regime. Using the toothpick inoculated method, the base sheath of wenmai 6 plant was inoculated with *R. cerealis* Rc207 at wheat tillering stage [33]. The sheath and/or stems of Wenmai 6 at five different infection time points (18, 36, 72, 96, and 240 hai) with *R. cerealis* Rc207 and in vitro mycelia were sampled for deep RAN-Sequencing. 

*N. benthamiana* plants were grown under standard glasshouse conditions at 25 °C with a 12-h light and 12-h dark regime.

The necrotrophic fungus *R. cerealis* Rc207, a highly aggressive strain collected in Northern China, was isolated in Shandong [7]. This strain Rc207 was cultured on potato dextrose agar (PDA) at 25 °C for 10 days before inoculation. 

All the primers and the sequences in the study are listed in Table S3.

### 4.2. Identification of Metalloproteases Genes in R. cerealis

The function of metalloproteases was annotated with the Hmmer software [34] based on the MEROPS database [20]. The BlastP with an E-value less than 1 × 10^−10^ was used to identify members of the candidate metalloprotease gene family from the *R. cerealis* Rc207 genome sequence. Signal peptide was used to predict using SignalP v4.0 [35], and putative proteins containing signal peptide were identified as secreted protein. Multiple alignments were determined with the DNAMAN 6.0.3.48 software (https://www.lynnon.com/download/ (accessed on 29 June 2022)). The expression profiles of metalloprotease family genes were drawn by the TBtools software [36]. A neighbor-joining (NJ) tree was constructed using the program MEGA (version 7.0) [37] based on the multiple sequence alignment of the 11 RcFL protein sequences in *R. cerealis* and 91 MEP36 proteins from other plant-pathogenic fungi (*B. sorokiniana* ND90P, *F. graminearum* PH-1, *F. pseudograminearum* CS3096, *P. oryzae* (P131, Y34, 70-15), and *R. solani* (AG1-IA, AG1-IB, 123E, AG-3 Rhs1AP, and AG-8) downloaded from the NCBI database (File S1).

### 4.3. RNA Extraction, cDNA Synthesis and Real-Time Quantitative PCR (RT-qPCR) Analysis

According to the manufacturer’s instruction, total RNAs from *R. cerealis* Rc207 in vitro mycelia and *R. cerealis*-inoculated sheaths and/or stems, or RcFL1-treated leaves of the susceptible wheat cv. wenmai 6 plants were extracted using the TRIzol reagent (Invitrogen, Life Technologies, Carlsbad, CA, USA) [38]. Reverse transcription was performed by using a PrimeScriptTM RT Reagent Kit with gDNA Eraser (Takara, City, Japan).

The *R. cerealis* infection process in wheat, fungal, or wheat RNAs were extracted for examining transcripts of the *RcFL1*, *TaChit3*, *TaChitIV*, and *TaCERK1* genes. Following the procedure described in Dong et al. [39], RT-qPCR was conducted with an ABI 7500 RT-PCR system (Applied Biosystems, Waltham, MA, USA). The relative expression of the target gene (*RcFL1*) of *R. cerealis* or the target genes (*TaChit3*, *TaChitIV*, and *TaCERK1*) of wheat were calculated using the 2^−ΔΔCT^ method [40], where wheat *Actin* gene (*TaActin*) and the *R. cerealis Actin* gene (*RcActin*) were used as the internal reference, respectively. Three independent biological replications were evaluated. 

### 4.4. Heterologous Expression of RcFL1 and Its MEP36 Domain RcFL1-MEP36 Peptides

The full encoding sequence of *RcFL1* and its partial sequence comprising the MEP36 domain were separately subcloned in fusion to the His-TF tag of the pCOLD TF vector, generating the expression vectors pHis-TF-RcFL1 and pHis-TF-RcFL1-MEP36. The DNAs of the resulting pHis-TF-RcFL1 and pHis-TF-RcFL1-MEP36 fusion constructs as well as pCOLD TF vector were each transformed into competent cells of *E. coli* BL21 (DE3), respectively. After these, *E. coli* BL21 cells were cultured in LB medium and induced with 0.5 mM isopropyl-β-d-thiogalactoside at 16 °C at 100 rpm overnight. The recombinant proteins were purified with Ni-NTA resin and eluted [41]. Finally, the SDS-PAGE (Bio-Rad, Hercules, CA, USA) was used to examine the purified proteins His-TF-RcFL1 and His-TF-RcFL1-MEP36 as well as His-TF control protein.

### 4.5. Cell Death-Inducing Activities and Disease Assays of RcFL1 Protein and Its MEP36-Containing Peptide RcFL1-MEP36

Cell death-inducing activities of the RcFL1 and RcFL1-MEP36 proteins of the heterologous expressed proteins (25 µL, 5 µM/L) were assayed by infiltrating samples into detached leaves from fully expanded secondary of two-month-old wheat plants or four-week-old *N. benthamiana* [24,38]. 

Following protein infiltration for six hours, leaves were inoculated in the same location with 50 µL mycelial suspension of *R. cerealis* Rc207. These leaves were placed in Petri dishes containing filter paper saturated with sterile distilled water and kept under a 16-h day/8-h night regime at 25 °C. Leaf lesions were calculated by length×width and photographed at 3 days post inoculation (dpi) with *R. cerealis* Rc207 [24,38]. Six leaves were performed in each experiment and repeated three times.

## 5. Conclusions

In this study, a total of 116 metalloproteases were identified from the whole-genome sequences of the wheat sharp eyespot *R*. *cerealis* Rc207 and were divided over 24 protein families, including 34 secretory metalloproteases with a predicted signal peptide. All the 116 metalloproteases genes were expressed at the late stages, 96, and 240 hai, of the fungal infection to wheat. Of 11 MEP36/fungalysin family metalloproteases, 10 MEP36 were predicted to be secreted proteins and nine encoding genes were expressed during *R. cerealis* infection process to wheat. Phylogenetic analysis suggested that these MEP36 metalloproteases in *R. cerealis* were closely-related to those of *R. solani* but were remote to those of *B. sorokiniana, F. graminearum, F. pseudograminearum,* and *P. oryzae*. Interestingly, an MEP36 metalloprotease gene *RcFL1* displayed a significantly upregulated expression during the infection process, and RcFL1 has been verified to function as a virulence factor of *R. cerealis* possibly through inhibiting host chitin-triggered immunity and chitinases. Thus, *RcFL1* likely is a promising gene resource for improving resistance of wheat and other crop plants to *R. cerealis* through host-induced gene silencing strategy. This is the first systematic inventory of whole-genome metalloproteases in *R. cerealis*.

## Figures and Tables

**Table 1:** Characteristics of 11 *MEP36* metalloprotease genes (*RcFLs*) in *R. cerealis*.

Gene Name	Gene ID	Coding Sequence Size (bp)	Amino Acid (aa)	Molecular Weight (kD)	Signal P	pI	Secreted Protein
*RcFL1*	Rc_11192.1	2313	770	86.13	Yes	5.22	Yes
*RcFL2*	Rc_05163.1	1998	665	74.02	Yes	5.20	Yes
*RcFL3*	Rc_00285.1	2268	755	82.70	Yes	6.02	Yes
*RcFL4*	Rc_01454.1	1788	595	63.38	Yes	6.57	Yes
*RcFL5*	Rc_01923.1	1806	601	67.94	No	6.61	No
*RcFL6*	Rc_07950.1	1800	599	64.53	Yes	5.51	Yes
*RcFL7*	Rc_08727.1	2124	707	77.70	Yes	5.99	Yes
*RcFL8*	Rc_08876.1	2346	781	85.65	Yes	5.68	Yes
*RcFL9*	Rc_09888.1	1800	599	63.83	Yes	6.07	Yes
*RcFL10*	Rc_12867.1	2346	781	85.62	Yes	6.68	Yes
*RcFL11*	Rc_13990.1	1833	610	66.94	Yes	5.27	Yes

## Data Availability

The data presented in this study are available on request from the corresponding author.

## Supplementary Materials

The supporting information can be downloaded at: https://www.mdpi.com/article/10.3390/ijms231810691/s1.
