# Supplementary figures and images for: Whole-Genome Metalloproteases in the Wheat Sharp Eyespot Pathogen Rhizoctonia cerealis and a Role in Fungal Virulence
Source: Int J Mol Sci. 2022 Sep 14;23(18):10691. doi: 10.3390/ijms231810691 (PMC9505970; doi:10.3390/ijms231810691)

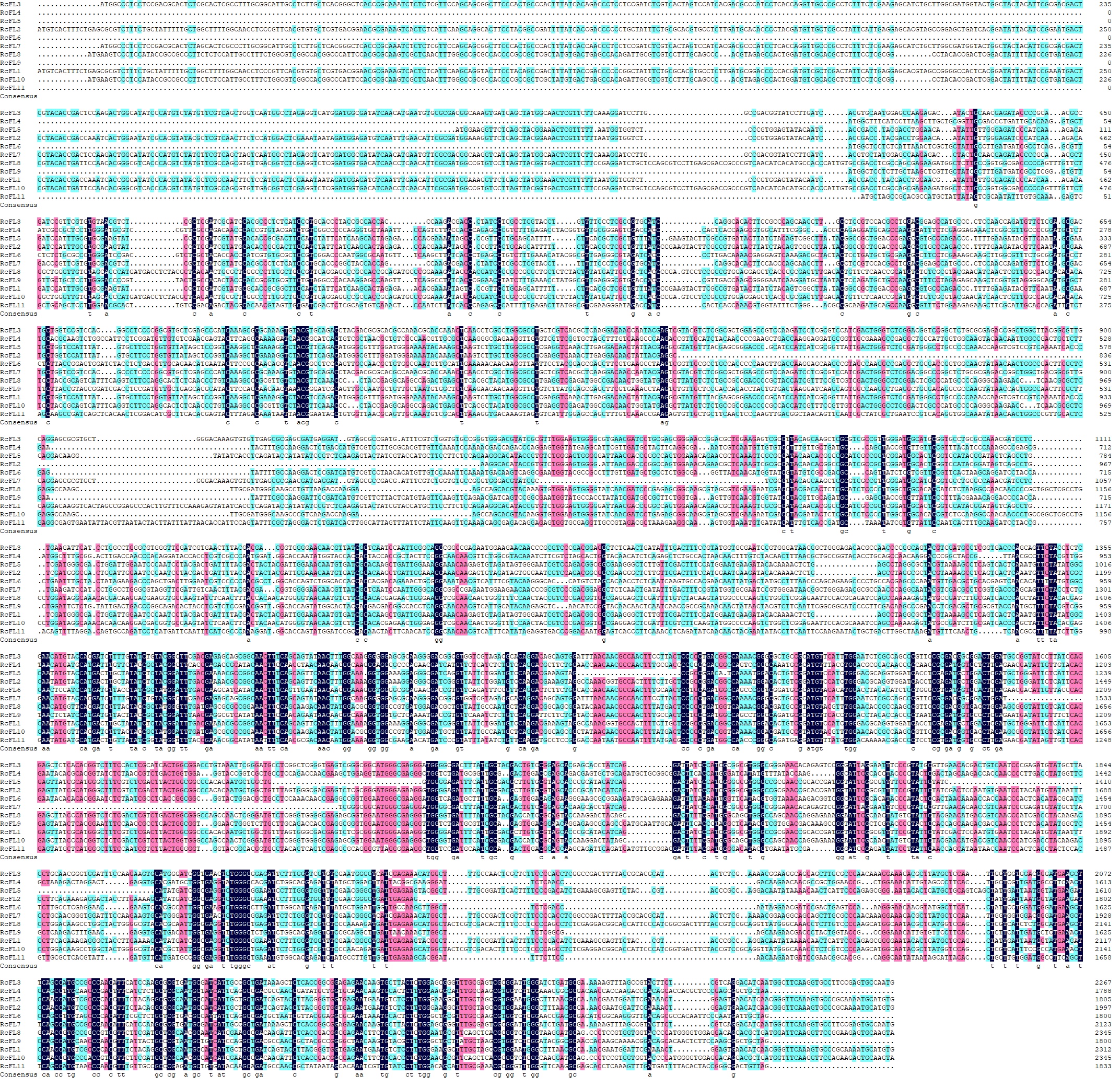

Supplement: Supplementary file 1 [file ijms-23-10691-s001.zip › Figure S1.jpg]

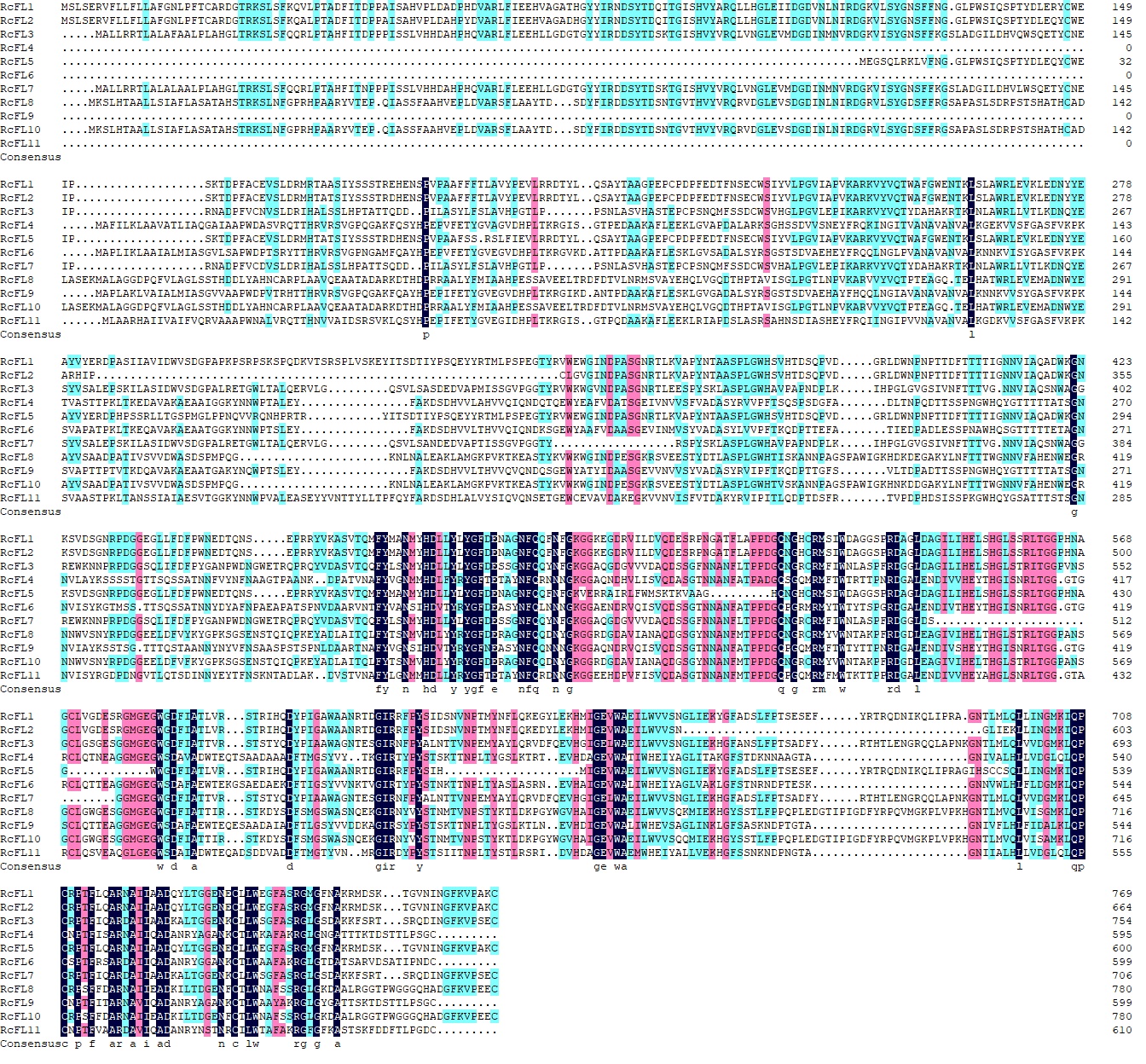

Supplement: Supplementary file 1 [file ijms-23-10691-s001.zip › Figure S2.jpg]

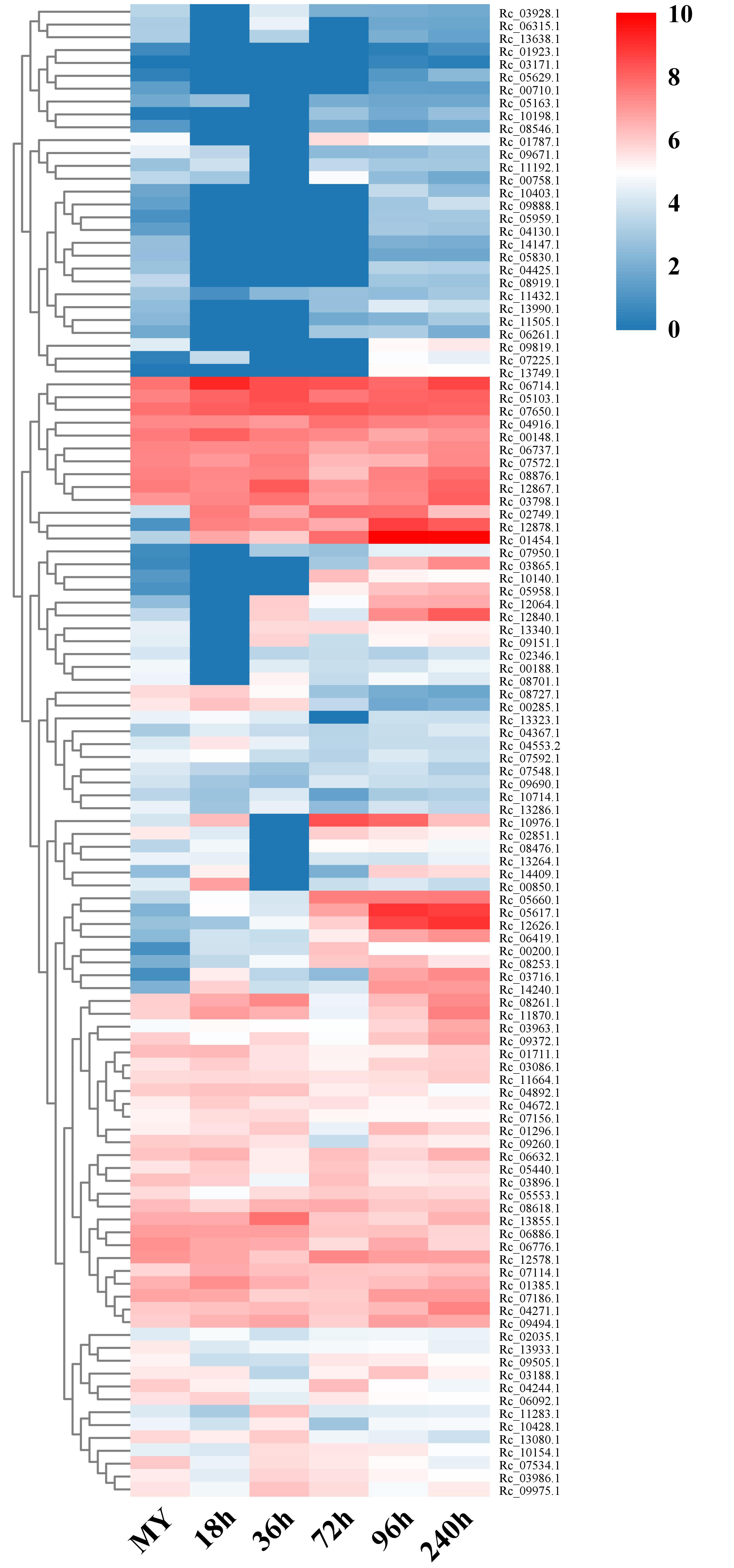

Supplement: Supplementary file 1 [file ijms-23-10691-s001.zip › Figure S3.jpg]

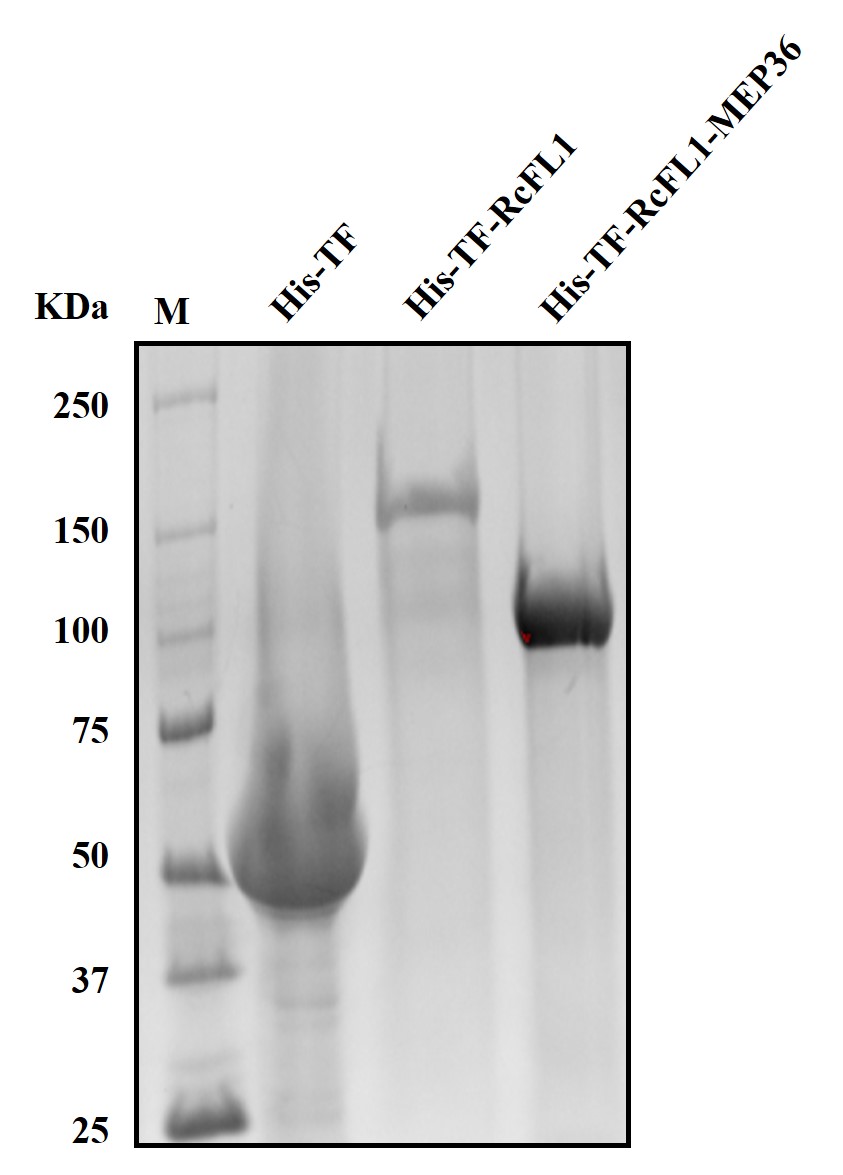

Supplement: Supplementary file 1 [file ijms-23-10691-s001.zip › Figure S4.jpg]
